# Impact of hemodialysis on P-wave amplitude, duration, and dispersion

**Published:** 2007-04-01

**Authors:** Abdenasser Drighil, John E Madias, Hanane El Mosalami, Nadia El Badaoui, Bahija Mouine, Wafae Fadili, Beenyouness Ramdani, Ahmed Bennis

**Affiliations:** 1 Department of Cardiology, Ibn Rochd University Hospital, Casablanca, Morocco; 2 Department of Nephrology, Ibn Rochd University Hospital, Casablanca, Morocco; 3 Mount Sinai School of Medicine of the New York University, New York, NY; 4 Division of Cardiology, Elmhurst Hospital Center, Elmhurst, NY

**Keywords:** P-wave duration, P-wave dispersion, hemodialysis, ECG, electrical impedance

## Abstract

Atrial fibrillation (AF) is a frequent arrhythmia in patients undergoing hemodialysis (HD). P wave duration (PWdu) and P wave dispersion (PWdi) have been shown to be predictors of emerging AF in different clinical conditions. We sought to study the impact of HD on PWdu, PWdi, and P wave amplitude in a cohort of patients undergoing HD. Seventeen patients (8 men, 31±10 years) were studied. Echocardiography parameters, the sum of the amplitude of P waves in all 12 ECG leads (SP), mean PWdu, and PWdi, along with a host of other parameters (body weight, heart rate, electrolytes and hemoglobin/hematochrit) were measured 1/2h, before and after, HD. SP increased (11.8±3.9 vs 15.3±4.0 mm, p = 0.004), mean PWdu remained stable (82.7±11.1 vs 81.6±10.5 ms, p = 0.606), PWdi decreased (51.7±19.1 vs 41.7±19.1 ms, p = 0.03), and left atrial dimension decreased (37.96±3.90 vs 30.62±3.38 mm, p = 0.0001), after HD. The change in PWdi correlated with fluid removed by HD (r = -0.55, p = 0.022). Re-measurements of P-wave parameters in a random group of 11 of the 17 patients revealed augmented SP (p = 0.01), and stable mean PWdu (p = 0.36), and PWdi (p = 0.31), after HD. Fluid removed by HD leads to an increase in SP, a stable mean PWdu, and decrease (or stability on re-measurement in a subgroup of patients) in PWdi. Stability of PWdu may be due to the effects of augmentation of the P-wave amplitude and the reduction of the left atrial volume, *cancelling* each other. Variability of PWdi may stem from the occasional impossibility to measure PWdu (or measure it correctly) in minute P-waves in certain ECG leads, which in turn profoundly affects the PWdi.

## Introduction

Atrial fibrillation (AF) is the most common sustained arrhythmia encountered in clinical practice. Epidemiological surveys have found that age, male gender, ischemic heart disease, hypertension, heart failure, valvular heart disease, diabetes, and disorders of the thyroid, lung, and pleura are independent risk factors for the development of acute AF. AF is also a highly prevalent (estimated ~13%) arrhythmia in haemodialysis (HD) patients, and is associated with a high mortality rate [1]. The re-entrant nature of AF requires areas of conduction delay to initiate and sustain the arrhythmia [2,3].  It has been shown that prolonged P-wave duration (PWdu) reflects such an electrophysiological substrate, which can be used as a predictor of AF development in various clinical settings [2,3]. Also it has been reported that P-wave dispersion (PWdi), because of its relation to both intra-, and inter-atrial inhomogeneous, interrupted conduction of sinus nodal impulses, is a noninvasive predictor of AF risk [4,5]. The consequences of HD on PWdu and PWdi have not been unequivocally documented and understood, and may be complex. In our previous work [6], we demonstrated that P-wave amplitude increased after HD and this correlated with augmentation in QRS amplitude; however, PWdu and PWdi remained *stable* after HD, which was in contradiction with the studies of some other authors, who reported *increase* [7,8], or *decrease* in these P-wave parameters. These, presumably electrophysiologically-mediated increases in PWdu and PWdi [7-9], should be considered along with the recently described alterations in the amplitude of P-waves [9], and stability of PWdu and Pwdi during  perturbations of various edematous states, attributed to extracardiac mechanisms [6,10,11]. Stability of PWdu was also reported with alleviation of another edematous state (congestive heart failure) [12], while others reported *shortening* of PWdu in patients with the same condition responding to diuresis [13]. Thus, it is possible that the response of PWdu and PWdi to HD is variable and it may be mediated by an interplay, of varying proportions, between electrophysiological influences and extracardiac mechanisms due to alleviation of fluid overload [14]. In the context of this controversy, we aimed at restudying the impact of HD on PWdu, PWdi, and P amplitude, and to analyze the determinants of their change, implementing some methodological enhancements to ensure greater accuracy of measurements of P-wave attributes.

## Methods

### Patients

Seventeen patients with end stage renal failure attending a routine midweek HD session were recruited in this study, after informed consent was obtained. All were receiving twice weekly bicarbonate based HD sessions lasting between 4 and 5 hours, using polysulfone capillaries and bicarbonate dialysate containing 138 Na^+^, 2.0 K^+^, 1.75 Ca^++^, and 0.5 Mg^++^ mmol/l. All HD sessions were uncomplicated. Informed consent was obtained from all participants of our study; the investigation was approved by the heads of the Departments of Cardiology and Nephrology. Although our study is considered research, it represents a low risk investigation, and thus it is exempt from the requirements of the federal regulations concerning IRB review and approval. (NIH document on "Human participant protections education for research teams" [45 CFR 46.101(b)], page 43).

### Study variables

Information pertaining to history, demographic data, and routine drug regimen were considered as study variables. Before and after HD the patients were weighed, had their blood pressure and heart rate obtained, had echocardiography, had a 12-lead ECG recorded, and a blood specimen was drawn for measurement of plasma electrolytes, and hemoglobin (Hb) and hematocrit (Ht). In addition to the fluid volume removed (FVR) by HD, the estimated fluid volume lost (FVL) was calculated by subtracting 500 cc from the FVR, to account for the patients' oral intake during HD.

### Electrocardiography

ECGs were recorded using a Heart Screen HF 112D (Innomed medical, Hungary) electrocardiograph at paper speed of 25mm/s and 50mm/s, and with standardization of 10 mm and 20 mm = 1.0mV; the ECG recordings at double speed and standardization were used for measurements. To ensure reproducibility of the ECGs before and after HD, the V1-V6 leads were obtained from fixed chest landmarks made with a skin marker.

### ECG measurements

**Figure 1:**
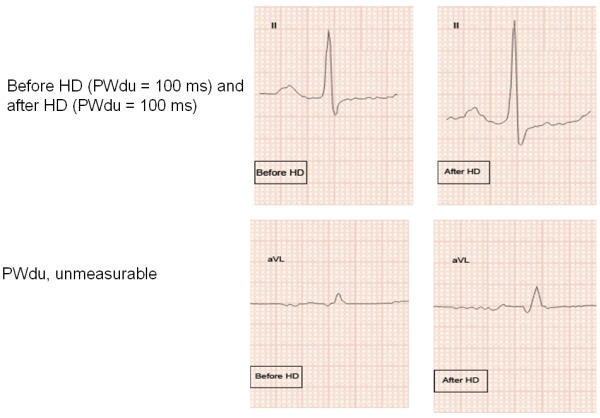
P-wave and QRS complex from leads II and aVL before and after HD; note the increase in the amplitude of QRS complexes in both leads following HD, and a slight increase in the amplitude of P-wave in lead II. PWdu did not change in lead II, while PWdu in aVL was not measurable either before or after HD, since the P-wave could not be differentiated from "noise".

The PWdu was measured in all leads from the first electrical activity to the offset at the junction between the end of P-wave deflection and the isoelectric line. Mean PWdu was the average of PWdu measured in all 12 leads. PWdi was calculated as the difference between the maximum and the minimum PWdu. Amplitude of P-waves in mm were measured to the nearest 0.25 mm from peak to nadir; P waves <1.0 mm were set at the fixed levels of 0.25, 0.5, and 0.75 mm. The 0.25 mm amplitude designation was considered when a P-wave consisted of a tiny perturbation in the isoelectric line prior to the QRS complexes; the 0.5 mm measurement was considered for the amplitude of P-wave estimated as such; the 0.75 mm measurement was considered when the amplitude of P-wave was >0.5 mm, but <1.0 mm. Occasionally the amplitude of P-waves was indistinguishable from the baseline (either isoelectric or non-isoelectric due to electronic noise) and thus was non measurable; also occasionally PWdu could not be measured in certain ECG leads with non measurable P-waves (Figure 1), or even in cases where the amplitude of P-wave was considered as measuring 0.25 mm, but still PWdu could not be discerned. The sum of the P-waves from all 12 ECG leads (SP) was then calculated, reflecting the changes in the amplitude of P-waves in all 12 ECG leads. Percent change (∆%) in the SPs, PWdu, and PWdi from the pre-HD values, were also used as variables. The QRS complexes in all 12 ECG leads were measured from peak to nadir in mm to the nearest 0.5 mm, and the sum (SQRS) was calculated. Sokolow-Lyon voltage (sum of the amplitudes of S-wave in V1 and R-wave in V5, or V6 >3.5 mV), and sex-specific Cornell voltage (sum of the amplitudes of S-wave in V3 and R-wave in aVL >2.8 mV in men, and >2.0 mV in women) were used as ECG criteria for left ventricular hypertrophy (LVH). Intra-observer variability was calculated as mean percentage error, derived from the difference between the two sets of measurements, divided by the mean of the 2 observations, and it was assessed in a randomly selected subset of 11of the 17 patients by repeating PWdu, PWdi, and SP measurements, before and after HD in all 12 ECG leads (Table 1) (Compare with the results of the first measurements on Table 3, which were used in the analysis). Re-measurements did not disclose any significant variability, except in the PWdi, which was found to be stable. The intra-observer variability for PWdi measurements was 6.3±2% before HD and 16.35±2.8% after HD; for mean PWdu it was 0.8±0.7% before HD, and 2.8±0.8% after HD; for maximum PWdu it was 1±1.4% before HD, and 7.7±1.0% after HD; for minimum PWdu it was 7.9±1.9% before HD, and 1.4±2.1 after HD; and for SP it was 8.5±1.5% before HD and 6.8±1.3% after HD.

### Echocardiography

**Figure 2:**
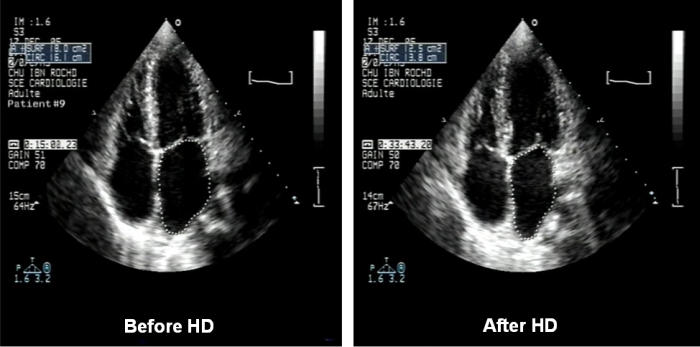
Echocardiogram (4-chamber view) of a patient before (left panel) and after (right panel) HD.

**Figure 3:**
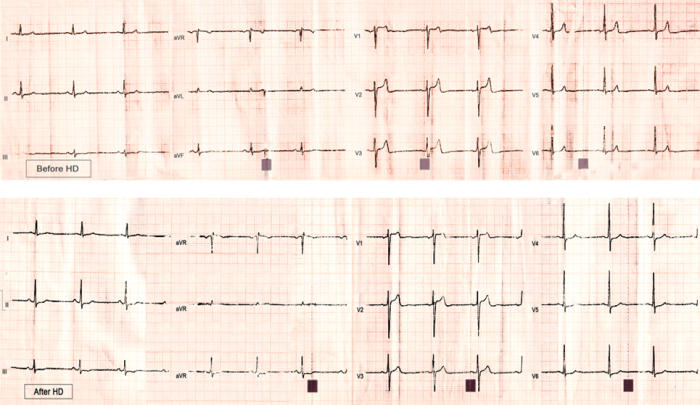
Standard 12-lead ECG (single standardization, and speed 25 mm/s) revealing post-HD augmentation of SQRS; P augmentation in this patient can be barely appreciated post-HD in leads II and III.

Two-dimensional echocardiography studies were performed ½ hour before HD, and were repeated ½ hour after HD, using a Phillips Sonos 5500 ultrasonographic machine equipped with a 3.5 MHz transducer. The same experienced echocardiographer performed all measurements. M-mode measurements were made by use of the American Society of Echocardiography leading-edge-to-leading-edge convention [15]. The following measurements of the left atrium (LA) [16] were obtained at end systole: 1) M-mode - derived anteroposterior linear dimension from the parasternal long-axis view, using 2-D guidance to position the cursor as described by the American Society of Echocardiography; 2) digitized planimetry of the LA cavity from the apical 4-chamber view (Figure 2); 3) LA volume derived from the M-mode dimension digital measurements, using a cube method, which assumes a spherical shape for LA. LA volume is: 4/3πr^3^, r=d/2, and d = M-mode anteroposterior dimension [17]. In addition LV end diastolic diameter (LVEDD), LV end systolic diameter (LVESD), LV end diastolic volume (LVEDV), LV end systolic volume (LVESV), and LV ejection fraction (LVEF) were measured before and after HD.

### Statistical analysis

Data are reported as mean±SD. Analysis employed the student's t test for paired data to determine the significance of differences before and after HD. Pearson's correlation coefficient for linear regression analysis of ∆% of the study variables resulting from HD was used. P<0.05 was considered as statistically significant. The SPSS (version 11.5) and Origin statistical packages were used.

### Results

**Figure 4:**
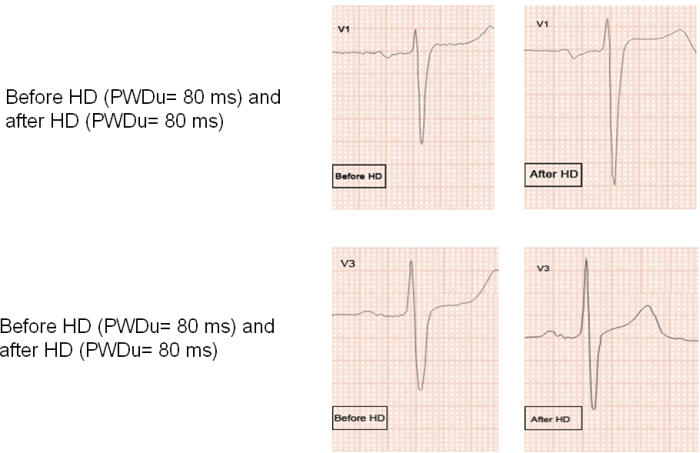
Augmentation in the amplitude of P-wave and QRS complex are evident in this patient post HD.

The characteristics of the subjects are shown on Table 2. The changes in measured variables are shown on Tables 3,4,5. Following HD, significant changes in electrolytes, and heart rate were observed in association with a fall of the patients' weight by a mean of 2.70±1.04 Kg (0.5 to 5). Significant ECG and echocardiography changes (Tables 3-5) (Figures 1,2,3,4) precipitated by HD included an increase of SP by 34.48±32.10 % and SQRS by 25.48±20.20 %. After HD, there was significant reduction in LA dimension (Figure 2) and LV volumes. The LVEF remained stable before and after HD (Table 4).

PWdu remained stable and PWdi decreased after HD (Tables 3and5) (Figures 1,3and 4). There was no correlation between ∆% in PWdi and ∆% in SP (r = -0.067, p = 0.795), LA diameter (r =0.038, p =0.884), K^+^ (r = -0.265, p = 0.304), Hb (r = -0.224, p = 0.388), and Ht (r = -0.034, p = 0.896). ∆% in PWdi correlated with FVR adjusted by pre-HD weight (r = -0.561, p = 0.019), FVL adjusted by pre-HD weight (r = -0.490, p = 0.046), and diastolic blood pressure (r = -0.55, p = 0.022).

PWdu was non measurable in lead II in 2 patients, in lead III in 1 patient, in lead aVL in 8 patients, and in lead V3 in 1 patient. In the other ECG leads it was measurable in all cases. When PWdu was non measurable before HD, it was still non measurable after HD, in-spite the overall augmentation in the amplitude of P-waves. In all, 10 patients had at least one lead non measurable, and for the total group of 17 patients 0.70±0.68 (0-2) leads per patient were non measurable. That represented 5.8% of all ECG leads that were non measurable before and after HD.

∆% in SP correlated poorly with ∆% in LVEDD (r = -0.414, p = 0.099), LVESD (r =- 0.215, p = 0.406), LVEDV (r =-0.393, p = 0.119), and LVESV (r = -0.315, p = 0.203), LA diameter (r = -0.316, p = 0.217), LA volume (r = -0.379, p = 0.134), weight (r = -0.363, p = 0.153), K^+^ (r = -0.058, p = 0.825), Hb (r = 0.474, p = 0.055), Ht (r = 0.338, p = 0.185), FVL adjusted by pre-HD weight (r = 0.399, p = 0.113), FVR adjusted by pre-HD weight (r = 0.360, p = 0.155), and SQRS (r = 0.407, p = 0.104).

## Discussion

The results of the present study corroborate those reported in our previous work [6], in regards to the intensity of HD (mean loss of fluid 3.0 liters in the previous study, 2.7 liters in the present study), augmentation of SP (40% in the previous study, 34.5% in the present study), stability of the PWdu, and augmentation SQRS after HD; minimum PWdu *increased* and PWdi *decreased* in the present study after HD, and remained *stable* in our previous study, and in the re-measured data from the 11 patients of the present study. It is important that this congruence in results occurred in these studies with ECG data generated by standard ECG recordings [6], and double speed and standard ECG recordings in the present study. Maximum PWdu remained stable after HD and PWdi decreased in the present study, in contradiction with other works, which found an increase in maximum PWdu and PWdi, and stable minimum PWdu [7,9], and decrease in PWduhyloglossus, after HD. The issue became even more complicated by the re-measurement of the PWdu, which showed stability of all P-wave parameters (Table 1). There appears to be certainty about the augmentation of the amplitude of QRS complexes [6,18-21], and of the P-waves [6,10], after HD, shown in many studies, our previous work, and the present study. However the response of mean, maximum, and minimum PWdu and in turn of PWdi to HD continues to remain elusive.

It is conceivable that this variability is due to: 1) the difference in the populations evaluated in different studies; 2) non-consideration of all the determinants affecting PWdu during HD; and 3) difficulties in measuring the PWdu, in general, or because of the methodologies employed.

In reference to the first, the mean age was 31 years in the present study, 42 years in our previous work [6], and  58 [7], 52 [8], and 54 [9], years in 3 other studies. Indeed, the incidence of AF (and by inference, of PWdu abnormalities) is known to increase, as patients get older, either in the general population [22], or in HD patients [1]. Taking into consideration that, in general, the population on HD is getting older, the relation between age and P-wave abnormalities is of significance. Accordingly in another study, the age of HD patients was the only independent predictor of PWdu [9]. The association of PWdu and age may suggest that age-related atrial conduction delay observed by some authors in healthy subjects [2], or patients with lone paroxysmal AF [23], may also be present in HD patients. Of course while delving in these issues, one should not lose track that the matter under consideration herein is not whether long PWdu is encountered in patients with before and/or after HD, but what is the *change* imparted by the procedure. Additionally, while only 17 % of our patients had hypertension, and none had ischemic heart disease or LA enlargement, in one of the above studies [7], the corresponding rates were 82 %, 64 %, and  50 %. All these factors are demonstrated to be strongly correlated with the occurrence of AF, independent of HD [24-26]. Of interest is that the above workers [7] found that PWdi increased only in patients with LA anteroposterior diameter >45 mm, and remained stable in those with diameter <45 mm. In our study, all patients had an anteroposterior diameter <45 mm. Although it has been stated that LA diameter is an important predictor of AF and that PWdu is related to LA diameter [5,27,28], other studies have reached contrary conclusions [4,29].

In reference to the second, many determinants of the *change* of the PWdu after HD, in addition to the changing electrolyte milieu, fluid volumes in different body compartments, atrial dimensions [7-9], may be operating, but not considered usually in the analysis. One in particular refers to the influence of the extracellular fluid volume reduction on the P-wave amplitude and PWdu. Accordingly loss of fluid with HD may impart an increase [14], or decrease in PWdu [9]. Such an *increase* has been attributed to the influence of extracellular fluid reduction, with resultant *increase* in the electrical impedance of the passive body volume conductor surrounding the heart, *augmentation* of the P-wave amplitude [10,30], and in turn increase in the PWdu, due to the inclusion in its measurement of an *earlier* onset and *later* offset [14]. This exact postulated mechanism has been also invoked in the increase in the QRS duration with alleviation of various edematous states [31], and artificial augmentation of the ECG voltage [32]. Loss of fluid in HD could also alleviate the hemodynamics, and stretch on the atria, thereby leading to beneficial changes in the electrophysiological substrate, and a decrease in PWdu [9]. The negative correlation found in the present study between ∆% in PWdi and fluid removed by HD probably reflects the impact of fluid overload on the P-wave parameters. However the highly statistically significant reduction of the LA diameter, area, and volume changes, noted after HD (Table 4), did not correlate with the the decreased PWdi. It is also conceivable that the interplay of the 2 above mechanisms, imparting *opposite* influences, may render an *unchanged* PWdu during HD, *and this is a key point of the present study*. However a corollary of the above is that depending on the proportion of the above 2 opposite influences in an individual patient or in a homogeneous patient population, an *increase* or *decrease* in PWdu, and in turn PWdi may be manifest, as shown in the literature [7-9].

In reference to the third, accuracy of the measurements of the PWdu and in turn PWdi is of paramount importance, and it is conceivable that differences in the methodology employed, have influenced the results of different studies. Single standardization with recording speed of 25 mm/sec but with enlargement by a factor of 3 on the photocopier of ECG printouts was used in one study [7], standard ECGs with inclusion of only V3 lead was employed in a second study [8], and filtered PWdu derived from signal-averaged ECG tracings was used in a third study [9]. In our previous work we used standard ECGs at a speed of 25 mm/sec [6], while we employed a double standardization and speed in the present study; this change in methodology has affected only the minimum PWdu, and the PWdi. However the different results in the re-measurement of the PWdu (Table 1), the problems in measurement encountered in some patients and in some specific ECG leads, particularly aVL (Table 5 and Figure 1) underscore the problems in employing PWdu and PWdi in patients undergoing HD. The difficulties in measuring PWdu when it is too small, encountered in particular leads is a serious impediment and may affect the accuracy of measurements. In the 11 patients, whose data were re-measured, we found that minimum PWdu did not change after HD (Table 1), while the initial measurement of all 17 patients disclosed an increase in minimum PWdu (Table 3), which in turn led to a decrease in PWDi in the initial assessment, and unchanged PWdi in the re-measurement. The increase in the minimum PWdu following HD is intuitively expected, since an augmented P-wave amplitude following HD would render the early part of onset and late part of offset measurable [14]. Nevertheless others have found an unchanged minimum PWdu and an increased maximum PWdu, and in turn an increased PWdi [7,8], which are counterintuitive as explained above. Finally in view of the difference in the response to HD of PWdu as assessed by the standard ECG [6-8], and the signal-averaged ECG [9], a comparison of the 2 technologies in the same patients is in order.

Several potential limitations of this study should be considered. Our patients were young and without vascular risk factors, commonly found in patients in HD. The young age of our patients is common in a developing country, like ours, where infection is the main etiology encountred in renal failure. Hence, the generalization of our finding to other populations of patients undergoing HD may be somewhat compromised. Advanced age, multiple risk vascular factors, long duration of HD, are frequent encounters in HD patients in developed countries. All these factors can influence the occurrence of arrhythmias during HD, and the P-wave parameters predicting them.

The most important finding of the present study and our previous work [6], is that in a total of 64 patients HD *per se* did not lead to any alteration of PWdu and PWdi (or perhaps a  questionable reduction in PWdi), which of course does not exclude the occasional  possible interaction between HD and other factors in resulting in such alterations in other patient populations. Finally the problems cited above in employing PWdu and PWdi in patients on HD, may explain the variable results in the literature, and suggest that exploring for a *uniform* response of the P-wave parameters in this setting, may be futile.

## Figures and Tables

**Table 1:**
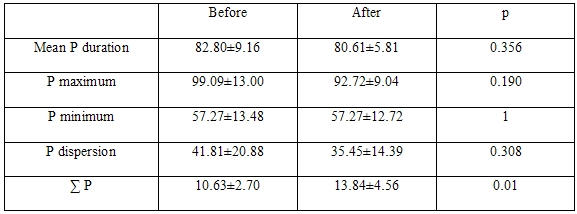
Results from Re-measurement of P-wave parameters (N = 11)

**Table 2:**
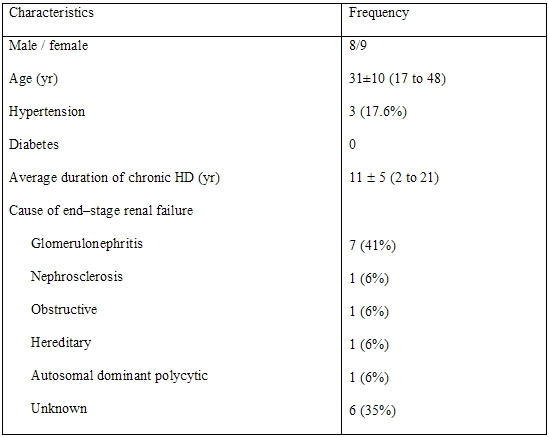
Baseline characteristics of patients (N = 17)

**Table 3:**
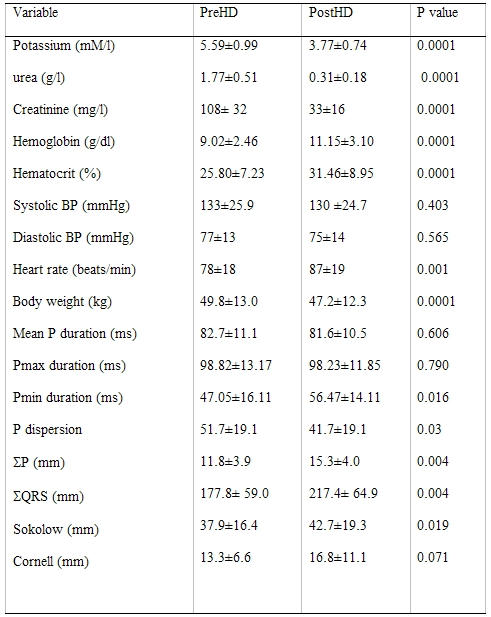
Study variables before and after hemodialysis (N = 17)

**Table 4:**
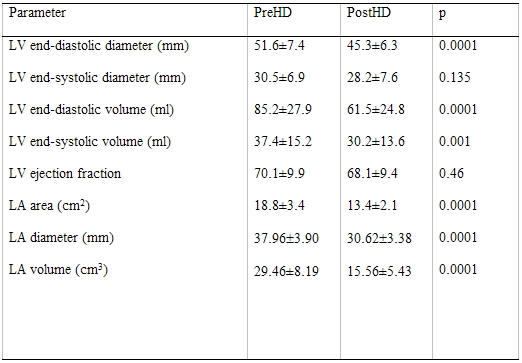
Echocardiographic parameters before and after hemodialysis (N = 17)

LA, left atrium; LV, left ventricle

**Table 5:**
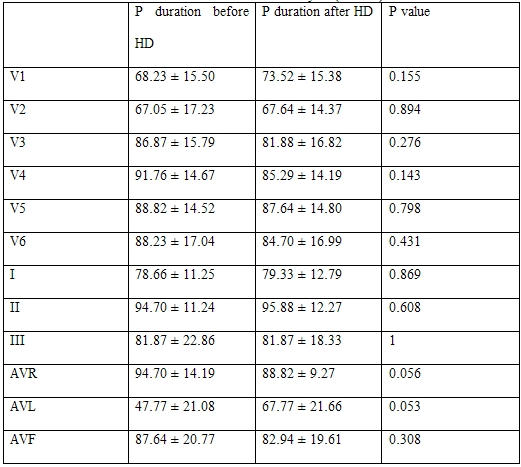
P duration before and after hemodialysis (N = 17)
